# Clinical validation of the Lumipulse G cerebrospinal fluid assays for routine diagnosis of Alzheimer’s disease

**DOI:** 10.1186/s13195-019-0550-8

**Published:** 2019-11-23

**Authors:** Maria João Leitão, Anuschka Silva-Spínola, Isabel Santana, Veronica Olmedo, Alicia Nadal, Nathalie Le Bastard, Inês Baldeiras

**Affiliations:** 1 Laboratory of Neurochemistry, Neurology Department, Centro Hospitalar e Universitário de Coimbra, 3000-075 Coimbra, Portugal; 2 Center for Neuroscience and Cell Biology, University of Coimbra, 3004-504 Coimbra, Portugal; 3 Dementia Clinic, Neurology Department, Centro Hospitalar e Universitário de Coimbra, 3000-075 Coimbra, Portugal; 4 Faculty of Medicine, University of Coimbra, 3000-548 Coimbra, Portugal; 5 Fujirebio, Iberia, S.I, Barcelona, Spain; 6 Fujirebio Europe N.V, Ghent, Belgium

**Keywords:** Alzheimer’s disease, Cerebrospinal fluid, Biomarkers, Chemiluminescent enzyme-automated immunoassay

## Abstract

**Background:**

Ongoing efforts within the Alzheimer’s disease (AD) field have focused on improving the intra- and inter-laboratory variability for cerebrospinal fluid (CSF) biomarkers. Fully automated assays offer the possibility to eliminate sample manipulation steps and are expected to contribute to this improvement. Recently, fully automated chemiluminescence enzyme immunoassays for the quantification of all four AD biomarkers in CSF became available. The aims of this study were to (i) evaluate the analytical performance of the Lumipulse G β-Amyloid 1-42 (restandardized to Certified Reference Materials), β-Amyloid 1-40, total Tau, and pTau 181 assays on the fully automated LUMIPULSE G600II; (ii) compare CSF biomarker results of the Lumipulse G assays with the established manual ELISA assays (INNOTEST®) from the same company (Fujirebio); and (iii) establish cut-off values and the clinical performance of the Lumipulse G assays for AD diagnosis.

**Methods:**

Intra- and inter-assay variation was assessed in CSF samples with low, medium, and high concentrations of each parameter. Method comparison and clinical evaluation were performed on 40 neurological controls (NC) and 80 patients with a diagnosis of probable AD supported by a follow-up ≥ 3 years and/or positive amyloid PET imaging. A small validation cohort of 10 NC and 20 AD patients was also included to validate the cut-off values obtained on the training cohort.

**Results:**

The maximal observed intra-assay and inter-assay coefficients of variation (CVs) were 3.25% and 5.50%, respectively. Method comparisons revealed correlation coefficients ranging from 0.89 (for Aβ40) to 0.98 (for t-Tau), with those for Aβ42 (0.93) and p-Tau (0.94) in-between. ROC curve analysis showed area under the curve values consistently above 0.85 for individual biomarkers other than Aβ40, and with the Aβ42/40, Aβ42/t-Tau, and Aβ42/p-Tau ratios outperforming Aβ42. Validation of the cut-off values in the independent cohort showed a sensitivity ranging from 75 to 95% and a specificity of 100%. The overall percentage of agreement between Lumipulse and INNOTEST was very high (> 87.5%).

**Conclusions:**

The Lumipulse G assays show a very good analytical performance that makes them well-suited for CSF clinical routine measurements. The good clinical concordance between the Lumipulse G and INNOTEST assays facilitates the implementation of the new method in routine practice.

## Background

Over the past few years, the path for both Alzheimer’s disease (AD) research and diagnosis has been radically changed due to developments in the field of biomarkers, as highlighted in the recent National Institute on Aging and Alzheimer’s Association biological definition of AD [1]. Different modalities of AD biomarkers have been implemented, including both neuroimaging and cerebrospinal fluid (CSF) biomarkers. In CSF, a combination of low levels of the 42-aminoacid isoform of amyloid beta (Aβ42) and high levels of total tau (t-Tau) and phosphorylated tau (p-Tau) is thought to reflect the two widely accepted pathophysiological hallmarks of AD: amyloid plaques and neurofibrillary tangles [2]. In clinical practice, these biomarkers are useful to detect or exclude AD, to make a prognosis at the Mild Cognitive Impairment (MCI) stage, and to guide patients’ management, particularly in atypical and clinically challenging cases [3, 4]. These biomarkers have also been incorporated in clinical trials, not only for patient selection—in fact, it was found in past AD drug trials that many individuals enrolled did not have AD brain pathology—but also to monitor target engagement and eventually as surrogate end points [5]. When an effective drug for AD is available, CSF biomarkers will become even more important in guiding the diagnosis and management of clinical cases.

However, the use of CSF biomarkers as diagnostic devices worldwide is hampered by problems of comparability of the results obtained in different centers or on different analytical platforms, low specificity towards non-AD cognitive diseases at the MCI stage, and limited understanding on how to interpret results, particularly if they seem discordant versus other biomarker modalities [6]. Until now, the INNOTEST enzyme-linked immunosorbent assays (ELISA) have been the mostly used assays for routine CSF biomarker analysis. These assays involve several manual pipetting steps, resulting in over 15% inter-laboratory variation of results, as reported in the Alzheimer’s Association international quality control program (www.neurochem.gu.se/TheAlzAssQCprogram) [7]. Another problem of these assays is the quite long turnaround time, as usually laboratories tend to accumulate samples over time, until they have enough to fill in an ELISA 96-well plate. Moreover, for the INNOTEST, some authors have reported an upward drift in Aβ42 values over time [8, 9].

Several international standardization initiatives have been launched to improve intra- and inter-laboratory variability, by standardizing pre-analytical variables, analytical protocols. and assay calibrators [10–12]. Although major advances have been made in the field [13], the situation is still not optimal, and universally accepted cut-offs have not been reached. To reduce variation in manual immunoassays and to cope with the increase in the number of referrals, multiplex assays and (semi) automated platforms have been developed [14–17]. Recently, four CSF analytes (Aβ42, Aβ40, t-Tau, and p-Tau) have been implemented on the fully automated Lumipulse G System, which is based on Chemiluminescent Enzyme Immunoassay technology. Lumipulse G uses single-analyte, ready-to-use, immunoreaction cartridges and renders quantitative results for an analyte within 30 or 35 min on the LUMIPULSE G1200 and G600II, respectively. These assays typically show an inter-laboratory variability of less than 10% (www.neurochem.gu.se/TheAlzAssQCprogram), but data regarding their clinical validation in research cohorts is still very limited [18–20]. Cut-offs that optimize the agreement between CSF biomarkers measured on the LUMIPULSE G600II instrument and amyloid imaging results by 18F-Florbetapir PET have been reported [21], but there are no validated cut-offs for these four CSF biomarkers in relation to clinical AD diagnosis.

The aims of this study were to (i) evaluate the analytical performance of the Lumipulse G β-Amyloid 1-42, β-Amyloid 1-40, total Tau, and pTau 181 assays on the fully automated LUMIPULSE G600II platform; (ii) compare CSF biomarker results of the Lumipulse G assays with the established manual ELISA assays (INNOTEST® β-AMYLOID_(1-42)_, INNOTEST β-AMYLOID_(1-40)_, INNOTEST hTAU Ag, and INNOTEST PHOSPHO-TAU_(181P)_); and (iii) establish cut-offs and the clinical performance of the Lumipulse G assays for AD diagnosis.

## Materials and methods

### Patients

All subjects included in this work are part of the Coimbra cohort [22, 23], recruited at the Neurology Department of Coimbra University Hospital, Coimbra, Portugal.

Patients were in a stable condition, without known acute comorbidities. A comprehensive diagnostic battery of tests was applied, including (1) cognitive instruments such as the Mini-Mental State Evaluation (MMSE) [24] Portuguese version [25], the Alzheimer Disease Assessment Scale-Cognitive (ADAS-Cog) [26, 27] Portuguese version [28], and a comprehensive neuropsychological battery with normative data for the Portuguese population (BLAD) [29] exploring memory (Wechsler Memory Scale sub-tests) and other cognitive domains (including language, praxis, executive functions, and visual-constructive tests); (2) standard staging scales which provide objective information about subject performance in various domains, including the Clinical Dementia Rating (CDR) [30] for global staging, the Disability Assessment for Dementia (DAD) [31, 32] for evaluation of functional status, and the Neuropsychiatric Inventory (NPI) [33, 34] to characterize the psychopathological profile, including the presence of depression. Patients also underwent a thorough biochemical, neurological, and imaging (CT or MRI and SPECT) evaluation. Positron emission tomography (PET) studies, using either [11C]-Pittsburgh Compound (PIB) or 18F-Florbetapir for amyloid imaging, were more restricted, although considered in younger patients, as previously described [35]. All the available information (baseline cognitive test, staging scales, clinical laboratory, and imaging studies) was used to reach a consensus research diagnosis, independently of the CSF biomarker results. AD patients were diagnosed according to the Diagnostic and Statistical Manual of Mental Disorders—fourth edition (DSM-IV-TR) criteria [36] and to the National Institute of Neurological and Communicative Disorders and Stroke-Alzheimer’s Disease and Related Disorders (NINCDS-ADRDA) [37]. To increase the strength of the clinical diagnosis of AD in patients that did not undergo amyloid PET (*n* = 45), a minimum 3-year follow-up was required.

The neurological control group consisted mainly of individuals that suffered from acute or chronic headaches, and a lumbar puncture (LP) was performed as part of their routine diagnostic evaluation in order to exclude bleeding or inflammation; in some subjects, this procedure was considered in the investigation of a peripheral polyneuropathy. In both cases, the CSF cyto-chemical evaluation was normal and a major CNS disease was excluded. In their brief cognitive assessment, they showed no subjective cognitive complaints and were independent in their instrumental daily life activities and most of them were still professionally active.

### CSF analysis

CSF samples were collected between April 2012 and July 2017, as part of the subject’s routine clinical diagnosis investigation. Pre-analytical and analytical procedures were done in accordance with previously proposed protocols [38]. Briefly, CSF samples were collected in 10-mL sterile polypropylene tubes (Sarstedt, Ref# 62.610.018), centrifuged within 2 h at 1800*g* for 10 min at 4 °C, aliquoted into 2-mL polypropylene tubes (Sarstedt, Ref# 72.694.007), and stored at − 80 °C until analysis. Storage time before biomarker analysis was between 7 and 77 months.

Samples were analyzed for the four markers (Aβ42, Aβ40, t-Tau, and p-Tau) by both assays (INNOTEST and Lumipulse) between November 2017 and September 2018. For each marker, both assays were performed in the same day, using the same aliquot. On the day of the analysis, samples were thawed at room temperature and the tubes were vortexed for 5–10 s. For INNNOTEST (INNOTEST β-AMYLOID_(1-42)_, INNOTEST β-AMYLOID_(1-40)_, INNOTEST hTAU Ag, and INNOTEST PHOSPHO-TAU_(181P)_, Fujirebio Europe, Ghent, Belgium), the four markers were measured separately, in duplicate, as previously described [39]. For Lumipulse, the four markers were quantified directly from the storage tubes using the Lumipulse G β-Amyloid 1-42, β-Amyloid 1-40, total Tau, and pTau 181 assays by the LUMIPULSE G600II automated platform and following the manufacturer’s instructions. Quality control testing was performed at the beginning of each test day to ensure that all measured values of each control level (low, medium, and high) were within the target ranges. The same batch of reagents for each marker/assay was used throughout the method comparison study.

The results of the Lumipulse G β-Amyloid 1-42 presented here have been standardized according to a certified reference material developed by the International Federation of Clinical Chemistry and Laboratory Medicine as recommended by their working group for CSF proteins [40]. Briefly, values of the calibration standards of the Lumipulse G β-Amyloid 1-42 were adapted to the certified reference material (CRM) resulting in an adjustment of concentrations that was linearly proportional throughout all the range. The aim of standardization to CRM is to harmonize immunoassays of Aβ42 to make results comparable across different platforms.

For the assessment of intra- and inter-assay variation for Lumipulse G β-Amyloid 1-42, β-Amyloid 1-40, total Tau, and pTau 181 assays, three different CSF samples with previously known high, intermediate, and low concentration of each of the four analytes were used and four replicates of each sample were measured on 5 different days.

For method comparison between INNOTEST and Lumipulse, as well as for establishing cut-offs and evaluating diagnostic performance of Lumipulse assays, 120 CSF samples were used: 40 from neurological controls and 80 from patients with a strong clinical diagnosis of AD (follow-up ≥ 3 years and/or positive amyloid imaging). In order to validate the established cut-offs, a small validation cohort comprising 20 clinical AD and 10 neurological controls was also employed.

### Statistical analysis

Statistical analyses were done in SPSS (version 23.0) (IBM SPSS, Chicago, IL) and MedCalc (version 11.6) (MedCalc Software, Mariakerke). Normality of continuous variables was assessed by the Kolmogorov-Smirnov test. For normally distributed continuous variables, Student’s *t* test was performed to assess the statistical significance of the difference between means. When continuous variables did not show normal distribution, the Mann–Whitney *U* test was used. Group differences between categorical variables were examined using the *χ*^2^ test. Correlation between Lumipulse G and INNOTEST assays was assessed through Spearman correlation and Passing–Bablok regression analysis. The existence of systematic and proportional difference between the two methods was assessed through the 95% confidence intervals (CIs) of the intercepts (if they included or not 0) and slopes (if they included or not 1) of the regression equations. Diagnostic performance of Lumipulse assays for the CSF markers or their ratios to distinguish between AD patients and controls was assessed by means of a receiver operating characteristic (ROC) curve analysis. Optimal cut-offs were determined by maximizing the Youden index and sensitivity, and specificity was calculated. The ROC curves were compared according to the area under the curve (AUC) comparison method of DeLong et al. [41]. Overall percentage of agreement (OPA) between Lumipulse and INNOTEST results were calculated as the sum of participants classified as “positive” or as “negative” by both modalities over the total number of participants. OPA was calculated both for each individual marker and their ratios as well as for the overall CSF profile. In accordance with the National Institute on Aging and Alzheimer’s Association criteria [1], subjects were classified as having a CSF-AD profile when they showed a positive β-amyloid plaques marker (either reduced Aβ42 or reduced Aβ42/Aβ40 ratio) in combination with a positive marker of fibrillary tau (increased p-Tau concentration).

## Results

### Analytical performance of Lumipulse assays

Results for intra-assay and inter-assay coefficients of variation (CVs) for the four Lumipulse assays are shown in Table 1. Intra-assay CVs for low, medium, and high concentration CSF samples ranged from 1.9 to 2.8% for Aβ42, from 0.8 to 1.4% for Aβ40, from 0.9 to 3.5% for t-Tau, and from 0.7 to 1.1% for p-Tau. CVs for inter-assay variation were 2.1–3.3% for Aβ42, 3.6–5.5% for Aβ40, 0.8–4.8% for t-Tau, and 2.0–3.6% for p-Tau.

**Table 1:** Intra- and inter-assay variation of the four Lumipulse assays on CSF samples

	Mean concentration (pg/mL)	Intra-assay		Inter-assay	
		SD (pg/mL)	%CV	SD (pg/mL)	%CV
Aβ42					
Low	193	5.4	2.79	6.3	3.28
Medium	648	12.6	1.94	13.7	2.11
High	1056	30.0	2.84	33.2	3.14
Aβ40					
Low	3369	31	1.21	185	5.50
Medium	5519	26	0.82	230	4.17
High	12,307	105	1.35	444	3.61
t-Tau					
Low	223	7.3	3.25	10.7	4.79
Medium	673	6.3	0.93	9.7	1.45
High	992	12.9	1.30	7.9	0.79
p-Tau					
Low	24.7	0.21	0.82	0.48	1.96
Medium	45.6	0.52	1.12	0.99	2.17
High	206.4	1.36	0.66	7.38	3.58

*SD* standard deviation, *CV* coefficient of variation

### Method comparison between INNOTEST and Lumipulse assays

Baseline characteristics at the time of lumbar puncture of neurological controls and AD patients are presented in Table 2. The population age ranged from 42 to 82 years old in controls and from 49 to 88 years old in AD patients, the latter being slightly but significantly older (*p* = 0.013). The female-to-male ratio was similar in both groups, and the percentage of ApoE-ε4 carriers in AD patients was over 53%, considerably higher than what we have previously shown in a Portuguese control population [42]. As expected, CSF levels of Aβ42, t-Tau, and p-Tau (both INNOTEST and Lumipulse results) were different in the AD group compared with controls. No differences between groups were seen in relation to CSF Aβ40 levels. There were no differences in sample storage time between controls and AD patients (*p* = 0.137).

**Table 2:** Demographic, clinical, genetic, and biomarker data of the study population

	Controls (*n* = 40)	AD (*n* = 80)	*p* value
Gender (M/F)	15/25	35/45	0.513
Age (years)	62.2 ± 9.7	66.8 ± 9.2	0.013
MMSE	28.8 ± 1.9	18.9 ± 3.7	< 0.001
ApoE-ε4 (%)	–	53%	NA
Aβ42			
INNOTEST (pg/mL)	848 ± 232	479 ± 121	< 0.001
Lumipulse (pg/mL)	726 ± 280	415 ± 126	< 0.001
Aβ40			
INNOTEST (pg/mL)	7999 ± 3975	7918 ± 3456	0.772
Lumipulse (pg/mL)	7749 ± 3139	8474 ± 2847	0.105
t-Tau			
INNOTEST (pg/mL)	203 ± 92	598 ± 293	< 0.001
Lumipulse (pg/mL)	217 ± 92	650 ± 291	< 0.001
p-Tau			
INNOTEST (pg/mL)	38.1 ± 13.0	75.3 ± 28.6	< 0.001
Lumipulse (pg/mL)	30.6 ± 11.6	106.9 ± 48.0	< 0.001

Data are expressed as mean ± SD, except for gender that is expressed as number of males (M) and females (F) and ApoE that is expressed as percentage of ε4 carries

In the subgroup of AD patients that underwent amyloid PET imaging (*n* = 35), the test was visually rated as positive in all patients. These patients were similar to the remaining AD patients that did not perform amyloid PET (*n* = 45) in terms of gender distribution, MMSE, ApoE genotype, and biomarker data (data not shown; *p* > 0.05 for all parameters). However, as expected, they were younger at the time of LP (62.1 ± 7.2 vs. 70.4 ± 8.9 years old; *p* < 0.001).

**Fig. 1:**
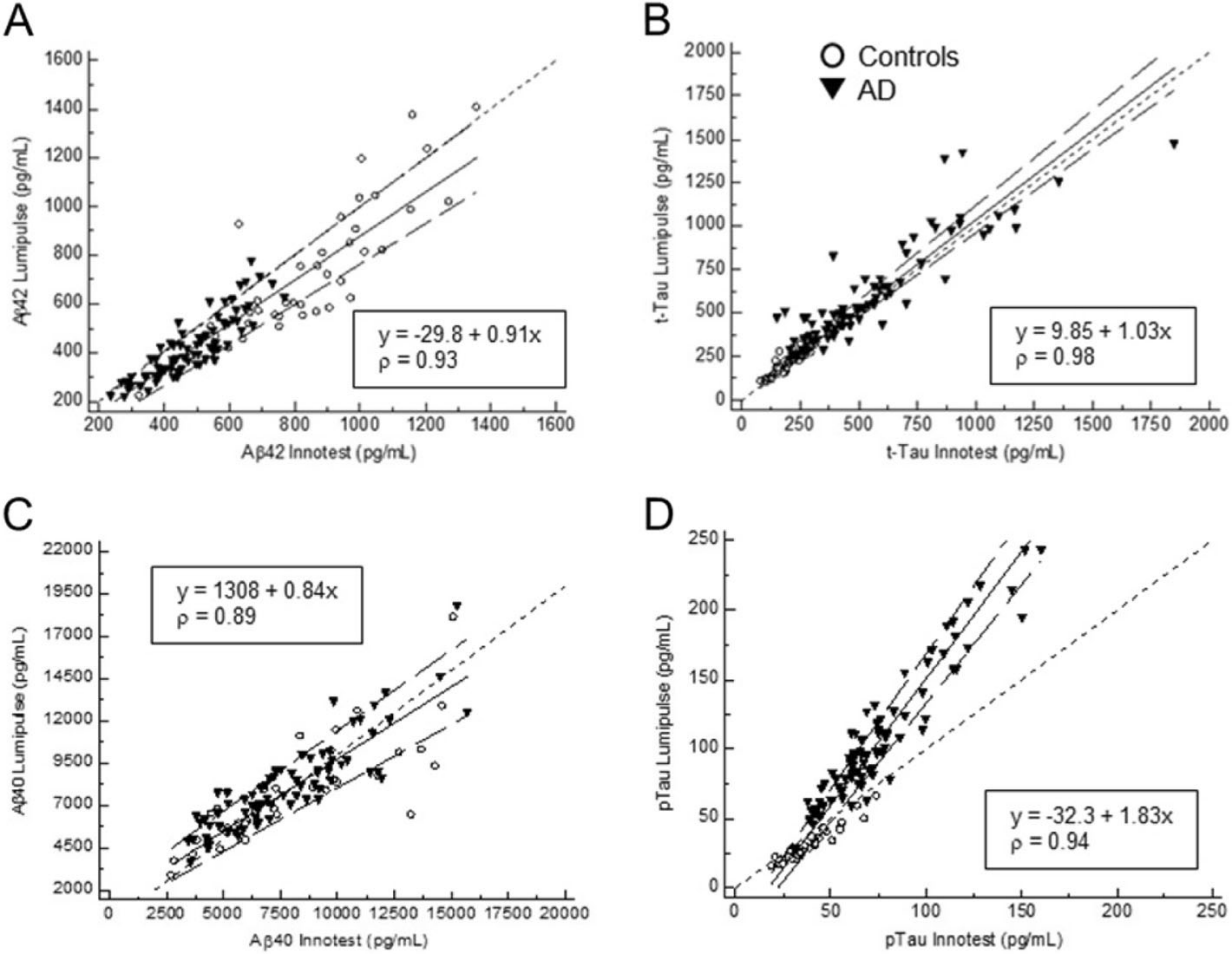
Passing–Bablok regression analyses comparing INNOTEST and Lumipulse results and conversion formulas for Aβ42 (**a**), t-Tau (**b**), Aβ40 (**c**), and p-Tau (**d**). Neurological controls are represented as open circles and AD patients as filled triangles

Passing–Bablok regression analyses comparing INNOTEST and Lumipulse results and conversion formulas for Aβ42, Aβ40, t-Tau, and p-Tau are shown in Fig. 1. The two methods showed strong correlations, with correlation coefficients [95% CI] of 0.93 [0.90; 0.95] for Aβ42, 0.98 [0.98; 0.99] for t-Tau, and 0.94 [0.90; 0.96] for p-Tau, with a slightly inferior correlation for Aβ40 (0.89 [0.82; 0.94]). For Aβ42 and t-Tau assays, the two methods did not show a systematic difference as indicated by the 95% CIs of the intercepts that included 0 (− 29.8 [− 6.96; 7.56] for Aβ42 and 9.85 [− 6.60; 27.35] for t-Tau), whereas a systematic difference was seen for Aβ40 (intercept = 1308 [599; 1997]) and p-Tau (intercept = − 32.3 [− 39.3; − 26.28]). Also, a proportional difference between the two methods was found for Aβ40 (slope = 0.84 [0.73; 0.94]) and p-Tau (slope = 1.83 [1.72; 1.95]), as indicated by the 95% CIs of slopes that did not include 1, but not for t-Tau (slope = 1.03 [0.97; 1.09]) and Aβ42 (slope = 0.91 [0.83; 1.01]).

### Diagnostic performance of Lumipulse assays

**Fig. 2:**
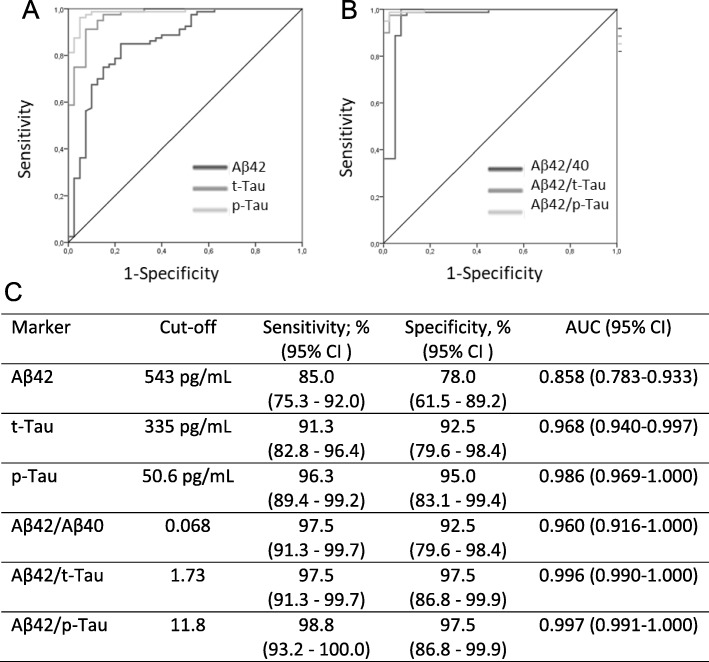
Receiver operating characteristics (ROC) curves for Aβ42, t-Tau, and p-Tau (**a**) and for the ratios between Aβ42 and Aβ40, t-Tau, and p-Tau (**b**) to distinguish between AD patients and controls. In the table (**c**), the cut-off values, sensitivity (in percentage), specificity (in percentage), and area under the curve (AUC) with the respective confidence interval (95% CI) derived from the ROC curves for each marker or ratio are depicted

We used ROC analysis to establish cut-offs for the different CSF markers and their ratios measured by Lumipulse to distinguish between AD patients and controls. As displayed in Fig. 2, the optimal cut-offs determined were 543 pg/mL for Aβ42, 335 pg/mL for t-Tau, and 50.6 pg/mL for p-Tau. p-Tau and t-Tau had optimal accuracy and showed an area under the curve (AUC) of 0.986 and 0.968, respectively, with sensitivity and specificity figures of 96.3%/95% for p-Tau and 91.3%/92.5% for t-Tau. Aβ42 also had a very good accuracy with an AUC of 0.858, 85.0% sensitivity and 78.0% specificity. Diagnostic performance was not assessed for Aβ40 alone, as this marker did not show statistical significance between AD and controls (see Table 1) and the ROC had an AUC < 0.700. Ratios between Aβ42 and the other markers were also assessed, and the following cut-off values were established: 0.068 for Aβ42/Aβ40, 1.73 for Aβ42/t-Tau, and 11.8 for Aβ42/p-Tau. The combination of Aβ42 with a second marker (Aβ40, t-Tau, or p-Tau) resulted in significant increases of accuracy in all cases, with AUCs significantly higher than those of Aβ42 alone (*p* < 0.05 for Aβ42/Aβ40 vs. Aβ42, *p* < 0.001 for Aβ42/t-Tau vs. Aβ42, and *p* < 0.001 for Aβ42/p-Tau vs. Aβ42). The comparison of the AUC of the ROC curves for the three ratios did not show a significant difference (Aβ42/Aβ40 vs. Aβ42/t-Tau: *p* = 0.083; Aβ42/Aβ40 vs. Aβ42/p-Tau: *p* = 0.090; Aβ42/t-Tau vs. Aβ42/p-Tau: *p* = 0.770).

We repeated the ROC analysis now using only AD patients with a positive amyloid imaging result (data not shown). Very similar cut-off values for distinguishing this subgroup of AD patients from controls were obtained: Aβ42 = 543 pg/mL, t-Tau = 336 pg/mL, p-Tau = 50.6 pg/mL, Aβ42/Aβ40 = 0.068, Aβ42/t-Tau = 1.73, and Aβ42/p-Tau = 10.7. Moreover, the AUCs as well as the sensitivity and specificity figure for each curve were also similar to the ones obtained for the entire AD group (Aβ42: AUC = 0.883, sensitivity = 91.4%, specificity = 77.5%; t-Tau: AUC = 0.959, sensitivity = 97.1%, specificity = 85.0%; p-Tau: AUC = 0.990, sensitivity = 97.1%, specificity = 95.0%; Aβ42/Aβ40: AUC = 0.961, sensitivity = 100%, specificity = 92.5%; Aβ42/t-Tau: AUC = 0.996, sensitivity = 100%, specificity = 97.5%; Aβ42/p-Tau: AUC = 0.999, sensitivity = 100%, specificity = 97.5%).

The cut-offs depicted in Fig. 2c were then applied to a validation cohort that comprised 20 clinical AD patients and 10 neurological controls. The demographic characteristics of this validation cohort, as well as the number of correctly classified individuals by the Lumipulse assays, are shown in Table 3. All control samples were within the normal range of all the assays or their ratios, whereas within the AD group, the percentage of samples with abnormal values ranged between 75 (for the Aβ42 assay) and 95% (for ratios between markers).

**Table 3:** Clinical performance of Lumipulse assays in a validation cohort

	Controls (*n* = 10)	AD (*n* = 20)	Total (*n* = 30)
Gender (M/F)	3/7	9/11	12/18
Age (years)	58.2 ± 10.7	67.2 ± 6.2	64.2 ± 9.7
Correctly classified			
Lumipulse			
Aβ42	10 (100%)	15 (75%)	25 (83%)
t-Tau	10 (100%)	18 (90%)	28 (93%)
p-Tau	10 (100%)	17 (85%)	27 (90%)
Aβ42/Aβ40	10 (100%)	19 (95%)	29 (97%)
Aβ42/t-Tau	10 (100%)	19 (95%)	29 (97%)
Aβ42/p-Tau	10 (100%)	19 (95%)	29 (97%)

Data are expressed as mean ± SD (age), as number of males (M) and females (F), and as the number (%) of correctly classified samples according to the previously determined cut-offs for Lumipulse assays

### Classification agreement between INNOTEST and Lumipulse

Using the Lumipulse cut-offs depicted in Fig. 2 and the previously described INNOTEST cut-offs [39, 43], concordance of biomarker results for the two methods was assessed for the three main markers separately, as well as for their ratios (Table 4). For Aβ42 and t-Tau, 95% and 97% of the samples, respectively, were concordant for biomarker abnormality between INNOTEST and Lumipulse, while for p-Tau the concordance was 88%. Percentage of concordant results for the Aβ42/Aβ40, Aβ42/t-Tau, and Aβ42/p-Tau ratios between INNOTEST and Lumipulse were 88%, 98%, and 94%, respectively. When combining biomarkers according to the NIA-AA criteria [1] into a CSF-AD biomarker profile (i.e., reduced Aβ42 or Aβ42/Aβ40 ratio in combination with increased p-Tau concentration), concordance was 90%.

**Table 4:** Agreement of INNOTEST and Lumipulse biomarker and ratio results on the training cohort

INNOTEST	Lumipulse		OPA
	Negative	Positive	
Aβ42			
Negative	41 (34.2%)	4 (3.3%)	114 (95.0%)
Positive	2 (1.7%)	73 (60.8%)	
t-Tau			
Negative	40 (33.3%)	0 (0.0%)	116 (96.7%)
Positive	4 (3.3%)	76 (63.3%)	
p-Tau			
Negative	34 (28.3%)	7 (5.8%)	105 (87.5%)
Positive	8 (6.7%)	71 (59.2%)	
Aβ42/Aβ40			
Negative	33 (27.5%)	10 (8.3%)	105 (87.5%)
Positive	5 (4.2%)	72 (60.0%)	
Aβ42/t-Tau			
Negative	39 (32.5%)	1 (0.8%)	117 (97.5%)
Positive	2 (1.7%)	78 (65.0%)	
Aβ42/p-Tau			
Negative	38 (31.7%)	5 (4.2%)	113 (94.2%)
Positive	2 (1.7%)	75 (62.5%)	
CSF-AD profile			
Negative	34 (28.3%)	8 (6.7%)	108 (90.0%)
Positive	4 (3.3%)	74 (61.7%)	

Data are expressed as the number of patients (percentage in relation to total). *OPA* overall percentage of agreement

Within the discordant results, Lumipulse was clearly more in agreement with the clinical diagnosis than INNOTEST for the p-Tau assay (the 7 patients classified as positive by Lumipulse and negative by INNOTEST all had a clinical AD diagnosis, while the 8 patients classified as negative by Lumipulse and positive by INNOTEST were all neurological controls) and the Aβ42/Aβ40 ratio (all 10 patients classified as positive by Lumipulse and negative by INNOTEST had a clinical AD diagnosis, while 4 out of the 5 patients classified as negative by Lumipulse and positive by INNOTEST were neurological controls). For the Aβ42/t-Tau, Aβ42/p-Tau, and CSF-AD profile, this was also true, as all patients with a positive Lumipulse and negative INNOTEST result were classified as clinical AD, whereas within patients with a negative Lumipulse and positive INNOTEST result, half were AD and half were controls. For the Aβ42 and the t-Tau assay, however, discordant results were not clearly in favor of either of the assays. Also noteworthy, within the 7 cases that had, according to the NIA-AA, a discordant CSF-AD profile, all of them had either p-Tau or the Aβ42/40 ratio in the border zone, i.e., within 10% of the cut-off in the pathologic direction [44].

## Discussion

Our results show that the Lumipulse G β-Amyloid 1-42, β-Amyloid 1-40, total Tau, and pTau 181 assays on the fully automated LUMIPULSE G600II platform have a very good analytical performance. In our hands, the inter-assay coefficients of variation ranged between 0.66 and 3.25%, while the intra-assay coefficients of variation varied between 0.79 and 5.50%. These values are in line with what was recently reported by Bayart and colleagues [20], are within what is desired for a routine diagnostic test, and are lower than what has been reported for INNOTEST and other ELISA assays, both by the manufacturer and by independent studies [45]. In addition to these analytical characteristics, Lumipulse assays also showed an excellent diagnostic accuracy for AD, reaching sensitivity and specificity levels from around 80% (in the case of Aβ42 alone) to up to more than 95% (for ratios between markers). These figures are at least as good as the ones generally reported for ELISA assays [46].

One of the main goals of this work was to establish cut-offs for the CSF biomarkers and their ratios, analyzed using the Lumipulse G platform, for the clinical diagnosis of AD. To the best of our knowledge, no other study has reported such cut-offs for all four biomarkers. The study by Alcolea and colleagues [21] included 94 participants from the Sant Pau Initiative on Neurodegeneration (SPIN cohort), but determined cut-offs for the Lumipulse assays by optimizing their agreement with 18F-Florbetapir PET amyloid imaging results, and not to the clinical diagnosis. Moreover, the population used was much more heterogeneous, including non-AD dementia cases. Therefore, the reported cut-offs of the three markers were quite different from ours. Interestingly, however, the cut-offs for the Aβ42/Aβ40 and Aβ42/t-Tau ratio were quite similar. The work of Paciotti and colleagues [19] compared AD (*n* = 42) and non-AD (*n* = 38) patients, assessing the diagnostic accuracy of only Aβ42 and t-Tau Lumipulse assay to distinguish between the two groups, but did not report the cut-off values. The recent work of Bayart and co-workers [20] used 44 AD patients and 42 controls to establish cut-offs for Lumipulse Aβ42 and t-Tau, but not for p-Tau or the Aβ42/40 ratio. These authors reached values of 437 pg/mL for Aβ42and 381 pg/mL for t-Tau, slightly different from ours, particularly for Aβ42. Apart from this small study, the only cut-offs for these assays that we are aware of and that were established based on clinical diagnosis are the ones recommended by the manufacturer. These were calculated based on the comparison of 60 probable AD patients and 43 non-demented controls (other neurological disorders such as psychiatric disorders, epilepsy, and multiple sclerosis), using a statistical approach similar to ours (ROC curve analysis with cut-offs selected based on maximal Youden index). Although slightly higher, the cut-offs for Aβ42, t-Tau, and p-Tau are not very different from ours (599 pg/mL, 404 pg/mL, and 56.5 pg/mL, respectively). The small differences between our cut-offs and previously reported ones could be attributed to the characteristics of the population or deviations in the pre-analytic protocol. In our control population, similarly to what is reported by Bayart et al. [20], we included cognitively normal patients with a suspicion of a neurological disease, but in whom a major CNS disease was excluded. However, while our control group includes mainly idiopathic headaches and some peripheral polyneuropathies, the control population that was selected by Bayart and colleagues is much more heterogeneous, including a large diversity of diagnosis. In relation to the control population used by the manufacturer, other non-neurodegenerative neurological diseases were included, and that could account for the differences in t-Tau and p-Tau cut-offs. Moreover, as shown in Table 2, our population is quite young, probably due to the fact that it comes from a specialized memory clinic, and that could also add to the differences in t-Tau and p-Tau levels [47]. The fact that we noticed the same trend for our INNOTEST cut-offs also argues for it being related to the population or pre-analytical confounders rather than the assays. Although the pre-analytical protocol that we used was similar to the one used by the manufacturer’s and by Bayart et al., there are slight differences, particularly in relation to the study of Bayart and colleagues, in relation to the type of tubes used for aliquoting, and filling of the tubes, that could justify the small variation in the cut-offs, particularly for Aβ42 [10, 48]. Noteworthy, our cut-off for the Aβ42/Aβ40 ratio is practically the same as the one recommended in the package insert (0.069), reinforcing the notion that this ratio is a more robust and easily standardized marker.

Although our study was limited by the relatively small sample size, a few points make us confident in the established cut-offs. First, when we re-calculated our cut-offs using only the subset of AD patients that had a confirmatory amyloid PET imaging result, the values reached were essentially the same as for the whole cohort. Second, the diagnostic accuracy derived from these cut-offs of both the Lumipulse assays and their ratios was at least as good as the one we have previously reported for the INNOTEST assays, employing larger cohorts of AD and neurological controls but with similar characteristic than the one included here [39, 43]. Moreover, we performed a small validation of our cut-offs in an independent cohort, which showed a good accuracy, correctly classifying 83% (for Aβ42 alone) to 97% (for ratios between markers) of the individuals. Interestingly, in this validation cohort, all controls were correctly classified by all markers and all three ratios performed exactly the same. If we compare the accuracy figures of this validation cohort (Table 3) with the values depicted in Fig. 2c for the discovery cohort, the total diagnostic accuracy is similar, although the data for the validation cohort seems in favor of the specificity. However, this validation population is very small, and further studies are needed to fully evaluate the accuracy of this cut-offs, ideally with a multi-center design.

As recently shown by others [20, 21, 49], a strong correlation between Lumipulse and INNOTEST Aβ42 and t-Tau assays was observed. Aβ40 and p-Tau also showed good correlations, in line with the work of others [20, 50, 51]; however, both a systematic and a proportional difference between methods was observed. Although both systems use similar antibody combinations, the discrepancy between absolute levels measured by both platforms might be due to differences in the technology. Furthermore, for Aβ40, the need to dilute samples in the ELISAs that induce an extra source of error might explain the slightly lower correlation coefficient for this assay. For p-Tau, although the correlation is good, in our cohort, Lumipulse p-Tau values seem to be higher than INNOTEST in the high range, and a bit lower in the low range. Nevertheless, the discrimination between AD and controls was better for the Lumipulse than for the INNOTEST.

In spite of differences in the absolute values for the various markers and ratios, both methods classified individuals in a similar way, with overall percentages of agreement of classification between 87.5 and 97.5%. Interestingly, in the majority of cases with discordant results in at least one of the biomarkers or their ratios (23 out of 29), Lumipulse classification was in agreement with the clinical diagnosis. Concordance analyses of biomarker abnormality based on cut-points are relevant to allow method comparisons on an individual level. When applying the NIA-AA criteria [1] to classify subject as having or not a CSF-AD profile, we observed that 12 cases (10%) were discordant according to the analytical method used. However, in all of these discordant cases, the marker that was discordant (either p-Tau or the Aβ42/Aβ40 ratio) had values near the cut-off that were within the usually called border zone [44]. Biomarker values near the cut-point need to be interpreted with caution, as technical or biological variation can influence the absolute values. Therefore, results within this border zone should be interpreted as at risk for abnormality rather than a conclusive positive or negative outcome.

One of the findings of our study was that the combination of Aβ42 with a second marker, either another amyloid marker (Aβ40) or a neurodegeneration/fibrillary tau marker (t-Tau or p-Tau), resulted in significant increases of accuracy for all cases, with the three ratios reaching a similar diagnostic accuracy. Therefore, our results confirm the superior value of the ratios and also highlight the use of the Aβ42/Aβ40 to compensate for individual differences in amyloid precursor protein processing that otherwise would result in an incorrect interpretation of Aβ42 CSF results [52]. Moreover, it has been shown that the CSF Aβ42/Aβ40 ratio can better predict abnormal cortical amyloid deposition compared with CSF Aβ42 [53, 54] and compensate for the effects of pre-analytical interfering factors, such as tube type, freeze/thaw cycles, and CSF volumes, therefore contributing towards pre-analytical standardization [55, 56]. Worth mentioning, in our analysis in the subgroup of 35 AD patients with positive amyloid imaging, three had a normal Lumipulse Aβ42 result, while the Aβ42/Aβ40 ratio was abnormal in all cases. Our results therefore support the use of the Aβ42/Aβ40 ratio in clinical care settings.

We believe that one of the main strengths of our study relies in the study design: the four AD CSF biomarkers (Aβ42, Aβ40, t-Tau, and p-Tau) were measured simultaneously, from the same aliquot, using both the Lumipulse and INNOTEST assays; the same batch of reagents for each marker/assay was used and a standard CSF pre-analytical procedure was followed throughout the study. In addition, the Lumipulse Aβ42 levels were standardized to the recently developed CRM, therefore allowing comparison with future studies. However, some limitations of the current study must also be addressed. In our study, some samples had been stored for quite a long time and this might have influenced the absolute levels of the different markers measured. However, a previous study has showed stable CSF Aβ42, t-Tau, and p-Tau concentrations over 12 years of biobank storage [57]. As participants in this study are part of a living cohort, neuropathological confirmation was not available, leaving the possibility of misdiagnosis. We tried to circumvent this problem by including only patients with a clinical AD diagnosis with a high degree of certainty, either due to a confirmatory amyloid imaging test or to their long clinical follow-up. A major limitation of this study is the small sample size. As explained above, by including only AD patients with a high degree of certainly, we limited the number of patients that could be included in the analysis. Moreover, as our population comes from a specialized memory clinic, having assess to neurological control samples is also a major difficulty. To address this extremely important issue of sample size, we propose that a multi-center study, including a large number of subjects and involving different laboratories that already have experience with both assays (Lumipulse and INNOTEST), should be conducted.

## Conclusion

Altogether, our results of an excellent diagnostic accuracy, allied to the fact that the automated assays reduce both intra- and inter-assay variability and reduce turnaround time, support the introduction of these assays in AD routine diagnostics, hopefully leading to more reproducible biomarker results worldwide.

## Data Availability

The datasets used and/or analyzed during the current study are available from the corresponding author on reasonable request.

## Abbreviations

Aβ42: 42-Aminoacid isoform of amyloid beta
Aβ40: 42-Aminoacid isoform of amyloid beta
AD: Alzheimer’s disease
ADAS-Cog: Alzheimer Disease Assessment Scale-Cognitive
ApoE: Apolipoprotein E
AUC: Area under the receiver operating characteristic curve
BLAD: Bateria de Lisboa para Avaliação de Demência (Lisbon Battery for Dementia Assessment)
CDR: Clinical Dementia Rating scale
CI: Confidence interval
CNS: Central nervous system
CSF: Cerebrospinal fluid
CV: Coefficient of variation
CT: Computed tomography
DAD: Disability Assessment for Dementia
DSM-IV-TR: Diagnostic and Statistics Manual for Mental Disorders—fourth edition
ELISA: Enzyme-linked immunosorbent assay
F: Females
LP: Lumbar puncture
M: Males
MCI: Mild Cognitive Impairment
MMSE: Mini-Mental State Examination
MRI: Magnetic resonance imaging
NIA-AA: National Institute of Aging-Alzheimer Association
NINCDS-ADRDA: National Institute of Neurological and Communicative Disorders and Stroke-Alzheimer’s Disease and Related Disorders
NPI: Neuropsychiatric Inventory
OPA: Overall percentage of agreement
PET: Positron emission tomography
PIB: [11C]-Pittsburgh Compound
p-Tau: Hyperphosphorylated Tau protein
ROC: Receiver operating characteristics
SD: Standard deviation
SPECT: Single-photon emission computed tomography
SPSS: Statistical Package for the Social Sciences
t-Tau: Total Tau protein
